# Insights into the Mechanisms of Chloroplast Division

**DOI:** 10.3390/ijms19030733

**Published:** 2018-03-04

**Authors:** Yamato Yoshida

**Affiliations:** Department of Science, College of Science, Ibaraki University, Ibaraki 310-8512, Japan; yamato.yoshida.sci@vc.ibaraki.ac.jp

**Keywords:** chloroplast division, mitochondrial division, endosymbiotic organelle, FtsZ, dynamin-related protein, glycosyltransferase protein

## Abstract

The endosymbiosis of a free-living cyanobacterium into an ancestral eukaryote led to the evolution of the chloroplast (plastid) more than one billion years ago. Given their independent origins, plastid proliferation is restricted to the binary fission of pre-existing plastids within a cell. In the last 25 years, the structure of the supramolecular machinery regulating plastid division has been discovered, and some of its component proteins identified. More recently, isolated plastid-division machineries have been examined to elucidate their structural and mechanistic details. Furthermore, complex studies have revealed how the plastid-division machinery morphologically transforms during plastid division, and which of its component proteins play a critical role in generating the contractile force. Identifying the three-dimensional structures and putative functional domains of the component proteins has given us hints about the mechanisms driving the machinery. Surprisingly, the mechanisms driving plastid division resemble those of mitochondrial division, indicating that these division machineries likely developed from the same evolutionary origin, providing a key insight into how endosymbiotic organelles were established. These findings have opened new avenues of research into organelle proliferation mechanisms and the evolution of organelles.

## 1. Introduction

**Figure 1:**
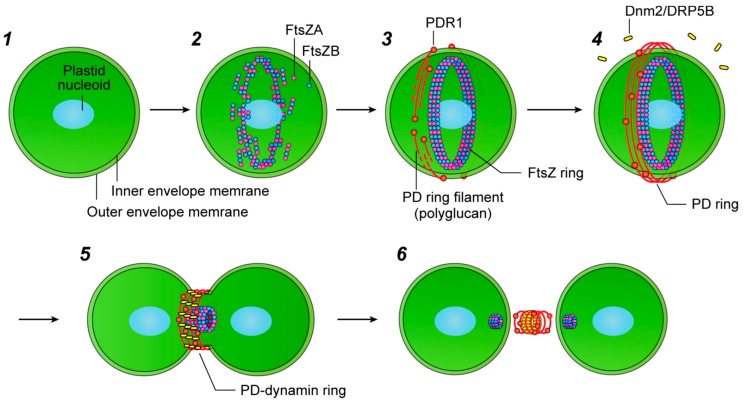
Representation of the plastid-division process. Plastid division occurs as follows: (**1**,**2**) Two types of FtsZ protein assemble in a heterodimer in the stromal region, then polymerize to form the FtsZ ring in the center of the plastid. To tether to the inner envelope membrane, FtsZ proteins bind to several membrane proteins. (**3**,**4**) PDR1 proteins attach to the outer envelope membrane above the site of the FtsZ ring, and it is hypothesized that PDR1 biosynthesizes polyglucan nanofilaments to form the PD ring from UDP-glucose molecules. (**5**) The GTPase protein Dnm2 (also known as DRP5B) binds to the PD ring filaments and is likely to generate the motive force for constriction. (**6**) Dnm2 proteins accumulate at the contracting bridge of two daughter plastids and pinch off the membranes. After the abscission of the plastids, the division machinery is disassembled. The inner PD ring and membrane proteins such as ARC6, and PDV2 are omitted from this representation. Modified from Yoshida et al. (2016) [21].

Chloroplasts (plastids) produce organic molecules and oxygen via photosynthesis, directly and indirectly providing a diverse array of living organisms with the materials they need to grow and develop. The activity of plastids over the past billion years has resulted in the dramatic greening of the Earth. Due to their endosymbiotic origin, plastids contain their own genomes and multiply by the binary fission of pre-existing plastids [1,2,3,4]. Although the mechanisms driving this division were long unclear, the discovery of a specialized ring structure at the division site of plastids in primitive unicellular alga provided groundbreaking insights into this process [5], and the rise of genomics and proteomics has further accelerated studies in this field. Consequently, some components for plastid division have been identified in the last 25 years [1,2,3,4,6]. It is now known that plastid division is carried out by a ring-shaped supermolecule termed the plastid-division machinery, which contains three or more types of ring structures: the plastid-dividing (PD) ring, which forms the main framework of the division machinery and comprises a ring-shaped bundle of nanofilaments on the cytosolic side of the outer envelope membrane of the corresponding organelle [4,5,7]; the FtsZ ring, a single ring constructed from homologs of the bacterial fission protein FtsZ located beneath the inner envelope membrane at the division site [8]; and the dynamin ring, a disconnected ring-like structure formed of a dynamin superfamily member on the cytosolic side of the outer envelope membrane at the site of organelle division (Figure 1) [9,10]. The importance of each component for plastid division has been well studied and summarized in detail elsewhere [3,11]; however, the functional mechanical details of the plastid-division machinery remain unclear. Various types of functional domains have been identified in the component proteins, including GTPase and glycosyltransferase domains, some of which are conserved not only within the plant kingdom but also in bacteria and non-photosynthetic eukaryotes; therefore, considerations and comparisons of these component proteins in other species will support our understanding of their fundamental functions during plastid division. Furthermore, it is now known that the mitochondrial- and peroxisome-division machineries carry out similar division processes to those of the plastids, suggesting that the elucidation of the plastid-division system will also provide insights into the proliferation of other membranous organelles within eukaryotic cells. This review summarizes and considers the domain architectures of the major proteins involved in plastid division, enabling the further exploration of the proliferation mechanisms in plastids as well as the proliferation mechanisms in mitochondria.

## 2. Structure and Assembly of the Plastid-Division Machinery

As mentioned above, plastids divide under the regulation of their division machinery, supramolecular complexes comprising a dynamic trio of rings, the PD ring, FtsZ ring, and dynamin ring, which span the plastid double membrane. Although the molecular function of each ring in the division machinery has not been fully revealed, studies using the alga *Cyanidioschyzon merolae* and the model dicot *Arabidopsis thaliana* have begun to uncover the molecular mechanisms by which this machinery functions. The formation of the plastid-division machinery is executed in a specific order during plastid division (Figure 1). As in bacteria, the assembly of the FtsZ ring in the stromal region is the first known event to occur at the plastid-division site, followed by the appearance of the inner PD ring beneath the inner envelope membrane at the division site. After the formation of the inner rings, the interaction between FtsZ and certain membrane proteins might transfer positional information about the FtsZ ring to the outside of the plastid, resulting in the binding of the glycosyltransferase protein PLASTID-DIVIDING RING 1 (PDR1) to the outer membrane, where it assembles the outer PD ring [12]. Another possibility was suggested following a series of electron microscopy (EM) observations in *C. caldarium*; small electron-dense vesicles were visualized along the putative division site before the assembly of the outer PD ring. Interestingly, the boundary of the vesicles appeared to coincide with one end of the PD ring filament, suggesting that these filaments might be biosynthesized on the surface of these vesicles using their components [5].

These inner and outer rings appeared to be linked to each other through nano-scale holes that appear on the groove of the division site, as revealed by scanning EM [13]. Recent yeast two-hybrid studies using several plastid-division proteins in *A. thaliana* showed that the FtsZ ring interacts with the inner membrane proteins ACCUMULATION AND REPLICATION OF CHLOROPLAST6 (ARC6) and PALAROG OF ARC6 (PARC6) in the stromal region [14,15]. ARC6 and PARC6 then further interact with the outer membrane proteins PDV2 and PDV1 in the intermembrane space to form the ARC6-PDV2 and PARC6-PDV1 complexes [15,16,17]. The intermembrane structure enables the translation of positional information regarding the FtsZ ring from the stromal region to the outer envelope membrane. In the final assembly step of the plastid-division machinery, dynamin-related protein (Dnm2/DRP5B) molecules cross-link the outer PD ring filaments. The sequence of assembly was confirmed in a range of knockdown/knockout experiments involving the plastid-division genes. When the expression of *PDR1* was downregulated, the FtsZ ring was assembled, but the Dnm2 proteins were not recruited to the division site [12]. Meanwhile, expression of a gene encoding the Dnm2 K135A mutant protein, which corresponds to a GTP-binding deficient mutant (K44A) of human dynamin 1, disturbed the formation of the dynamin ring and inhibited plastid division in *C. merolae*, despite the normal assembly of the FtsZ and PD rings [18,19,20]. Thus, neither FtsZ ring formation nor PD ring formation depends on the subsequent assembly of the plastid-division machinery, while the localization of Dnm2 relies on formation of the outer PD ring.

A series of ultrastructural studies provided structural insights into the contractile mechanism of the plastid-division machinery. Sequential EM observations of cells during plastid division revealed that the thickness of the inner PD ring does not change, but its volume decreases at a constant rate during contraction [22]. By contrast, the width and thickness of the outer PD ring monotonically increase during contraction in *C. merolae*, retaining its density and volume [22,23]. Similar EM observations of the outer PD ring changes have also been made in the green alga *Nannochloris bacillaris* [24], and in the land plants [25]. These morphological transitions of the outer PD ring during plastid division led to the idea that the outer PD ring filaments can slide and squeeze the plastid membranes. The establishment of a technique for isolating the intact plastid-division machinery from *C. merolae* cells provided a major breakthrough on this issue [13]; the isolated plastid-division machinery not only formed a circular structure, but also formed super-twisted and spiral structures featuring both clockwise and anticlockwise spirals. The existence of plastid-division machinery in these twisted states indicates the motive force involved in the contraction of plastid membranes; indeed, the plastid-division machinery autonomously contracted when plastid membranes were dissolved by detergent. In addition, reconstituted plastid FtsZ rings also displayed contractile ability. Although the detailed molecular mechanism involved is still unclear, the contraction process of the FtsZ ring accompanies the transition of the FtsZ protofilament from a less dynamic to a more dynamic state; therefore, this dynamic transition of protofilament states is assumed to induce a decrease in the average protofilament length in the FtsZ ring and thus cause contraction [21]. This led to the undertaking of complex studies to reveal how the plastid-division machinery is transformed morphologically during contraction, and which of its component proteins play a key role in generating this force.

## 3. The FtsZ Ring

**Figure 2:**
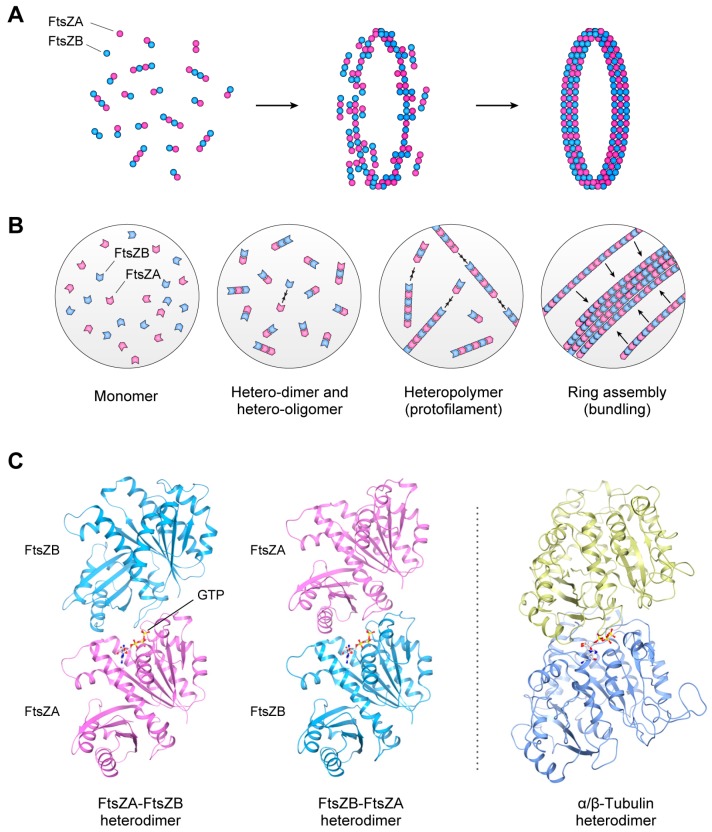
Assembly of the FtsZ ring. (**A**,**B**) FtsZ molecules assemble into hetero-oligomers, then these FtsZ protofilaments bundle and assemble into a ring structure in the stroma region. (**C**) The two types of FtsZs can assemble into heteropolymer structures via FtsZA-FtsZB and FtsZB-FtsZA hetero-interactions. The protein structure of the tubulin heterodimer (PDB: 1TUB) is also shown on the right. The protein structures of *A. thaliana* FtsZ2 (shown as FtsZA in the Figure) and FtsZ1 (FtsZB in the Figure) were obtained using homology modeling in the Modeller program [35], and structural data for each protein molecule were visualized using CueMol: Molecular Visualization Framework software (http://www.cuemol.org/). Reproduced and modified from Yoshida et al. (2016) [21].

Plastid FtsZ is a homolog of the bacterial fission protein FtsZ [8,26,27], and was the first factor found to be responsible for plastid division, assembling into a ring beneath the inner envelope membrane at the division site [22,28,29]. Both plastid and bacterial FtsZ proteins are composed of two functional domains, a GTP-binding domain at the N-terminal and a GTPase-activating domain at the C-terminal, which interact with the opposing terminal of other FtsZ proteins to form a polymer strand (Figure 2) [30,31]. Interestingly, whereas bacteria have one *FtsZ* gene in their genome, the plastid *FtsZ* gene underwent duplication and these duplicated loci, now present in the nuclear genomes, are widely conserved throughout photosynthetic eukaryotes [8,32,33]. Thus, the *C. merolae* genome contains two *FtsZ* genes, *FtsZ2-1* (*FtsZ2* in *A. thaliana*; FtsZA group) and *FtsZ2-2* (*FtsZ1* in *A. thaliana*; FtsZB group), for plastid division [27,32,34]. Phylogenetic studies have found that FtsZA is more ancestral [32], containing a conserved C-terminal core motif similar to that of bacterial FtsZ, which is assumed to interact with other plastid-division proteins to tether the FtsZ ring to the inner membrane [6,8]. The other FtsZ group, FtsZB, lacks the C-terminal core motif and probably evolved following the duplication of the original *FtsZ* gene or its precursor during the establishment of the plastids after the endosymbiotic event.

Although the mechanistic details of FtsZ ring assembly and dynamics remain unclear, recent studies using a heterologous yeast system revealed that the two types of plastid FtsZs spontaneously formed heteropolymers then assembled into a single ring that could generate contractile force in the absence of any other related proteins (Figure 2A,B) [21,36]. Interestingly, the plastid FtsZ heteropolymer had higher kinetic dynamics for protofilament assembly, mobility, and flexibility than either homopolymer, suggesting that the gene duplication of plastid *FtsZ* led to innovation in the kinetic functions of the FtsZ ring for plastid division [21]. The FtsZ heteropolymers are structurally similar to the microtubules comprised of α- and β-tubulin, suggesting the convergent evolution of functions in the plastid FtsZs and eukaryotic tubulins (Figure 2C). Consistent with molecular genetic studies using *A. thaliana*, the assembly of the plastid FtsZ ring in vivo is further promoted by several regulatory factors. ARC6 positively regulates the assembly of the FtsZ protofilament through interactions with the FtsZA molecules [6,8]. PARC6 also interacts with FtsZA, inducing the remodeling of the FtsZ protofilaments for ring formation with support from ARC3 [6,8,15]. Moreover, ARC3 and GIANT CHLOROPLAST1 (GC1) negatively regulate FtsZ ring assembly [37,38]; however, as these FtsZ-associated genes are not well conserved in the plant kingdom, another regulatory system with unknown factors might also coordinate the assembly of the FtsZ ring.

## 4. The Dynamin Ring

**Figure 3:**
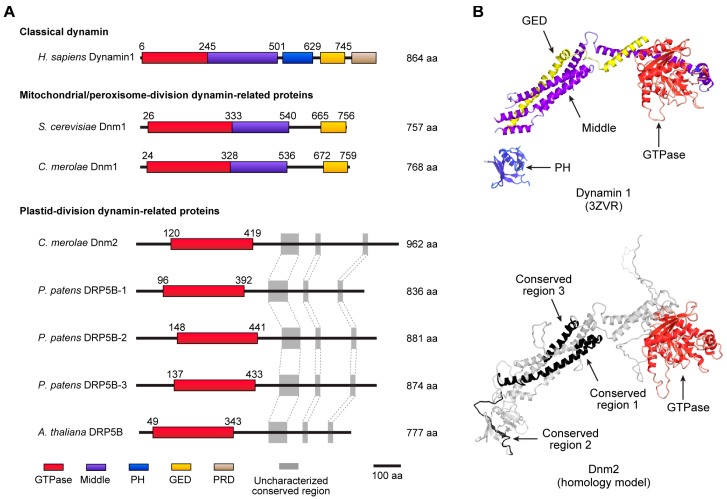
Structures of the dynamin superfamily. (**A**) Domain architectures of the dynamin superfamily. Dynamin 1 catalyzes clathrin-coated vesicle scission at the plasma membrane. Dnm1/DRP1 is involved in the division of mitochondria and peroxisomes. Dnm2 (also known as DRP5B/ARC5 in *A. thaliana* and moss) is involved in plastid division. GTPase domain (red); middle domain (purple); pleckstrin homology domain (PH, blue); GTPase effector domain (GED, yellow); and proline-rich domain (PRD, light brown). Domain architectures were identified using a conserved-domain search program [48]. (**B**) Protein structures of human dynamin 1 (classical dynamin) for vesicle scission and *C. merolae* Dnm2 for plastid division. The structure of dynamin 1 is represented with crystal structure data from an assembly-deficient dynamin 1 mutant, G397D ΔPRD (PDB: 3ZVR) [43], while the structure of Dnm2 is visualized using homology modeling based on the dynamin 1 structure. The functional domains in dynamin 1 are shown in red (GTPase domain), purple (middle domain), blue (PH domain), and yellow (GED domain); the proline-rich domain (PRD) is not shown. Uncharacterized conserved regions in Dnm2 are shown in black. The protein structure of Dnm2 was modeled as described in Figure 2.

A member of the dynamin superfamily, Dnm2 (also known as DRP5B or ARC5 in *A. thaliana*), is also observed in a ring structure at the plastid-division site (Figure 1) [9,10]. Originally, the classical dynamin protein was identified as a 100-kDa mechanochemical GTPase required for the scission of clathrin-coated vesicles from the plasma membrane [39,40]. Dynamin-related proteins are now known to be involved in diverse membrane remodeling events; for example, Dnm1 (also known as DRP1 in animals) is involved in mitochondrial and peroxisome divisions [40,41]. The dynamin superfamily proteins possess several functional domain subunits, including a large GTPase domain (approximately 300 amino acids), a middle domain, a pleckstrin-homology (PH) domain, a GTPase effector domain (GED), and a carboxy-terminal proline-rich domain (PRD) (Figure 3A,B, upper) [40,42,43]. Although the molecular mass of Dnm2 is similar to that of classical dynamin, a conserved domain search identified only the GTPase domain at its amino-terminal region (Figure 3A). The three uncharacterized regions occupying the rest of the sequence are highly conserved between Dnm2 proteins in plant species, implying that these regions might be responsible for key roles during plastid division (Figure 3A,B, bottom).

The functional importance of Dnm2 proteins during plastid division has been demonstrated in *C. merolae*, the moss *Physcomitrella patens*, and *A. thaliana* [9,10,44]. Dnm2 proteins formed a discontinuous ring structure at the plastid-division site in the early phases of plastid division, after which they appeared to link and assemble into a single ring [9,10]. Surprisingly, the plastid-division dynamin-related proteins are phylogenetically related to a group of dynamin-related proteins involved in cytokinesis [45]. Considering that the primitive algal genome encodes only two dynamin-related proteins, Dnm2 for plastid division and Dnm1 for mitochondrial/peroxisome division [34,46,47], these findings raise the question of the original function of the ancestral dynamin protein.

## 5. The PD Ring

In the 1980s and 1990s, EM observations revealed the presence of the PD ring at the division site of plastids in numerous photosynthetic eukaryotes [5,49]. Two (or three) types of electron-dense specialized ring structures comprise the PD ring: the outer PD ring is the main skeletal structure of the plastid-division machinery, and is composed of a ring-shaped bundle of nanofilaments, 5 to 7 nm in width, on the cytosolic side of the outer envelope membrane (Figure 1) [13,23]. In addition, an inner 5-nm-thick belt-like PD ring forms on the stromal side of the inner envelope membrane [22,50]. The outer and inner PD rings have been widely observed in the plant kingdom [5,49], while a third, intermediate, PD ring has only been identified in the intermembrane space of *C. merolae* and the green alga *N. bacillaris* [24,51]. Many angiosperms also form double rings at the constricted isthmi of dividing plastids, including proplastids, amyloplasts, and chloroplasts [5,49]. The PD ring structure can be observed only during the late phases of plastid division in land plants [49,52], suggesting that the number and electron density of the PD rings in these organisms might be too low to detect during the early phases of plastid division using EM, making their morphological dynamics more difficult to study in these species. Interestingly, a phylogenetic study of the PD ring identified a clear trend, in which the PD rings of the primitive unicellular eukaryotes with smaller genomes are larger than those of the multiplastidic cells of land plants [5,49]; therefore, *C. merolae* is one of the most suitable organisms for studying the involvement of the PD ring in plastid division. Based on these previous studies, the PD rings, especially the outer rings, were concluded to be universal across the plant kingdom, where they play an important role in plastidokinesis.

**Figure 4:**
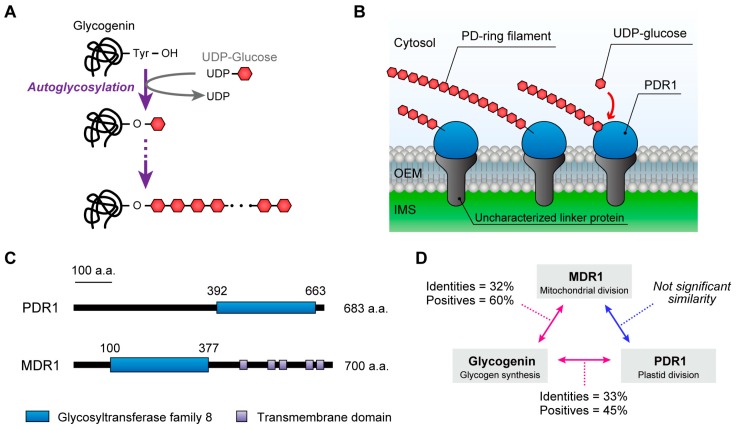

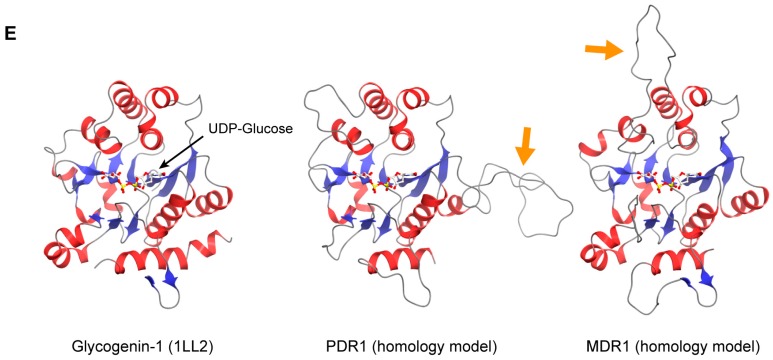
Working models of the glycosyltransferases glycogenin and PDR1. (**A**) Glycogenin is required for the initiation of glycogen biosynthesis, and can be autoglycosylated at a specific tyrosine residue to form a short oligosaccharide chain of glucose molecules to act as a priming chain for the subsequent biosynthesis of glycogen. (**B**) A schematic representation of PD ring filament biosynthesis by PDR1. A series of results suggested that PD ring filaments are composed of both PDR1 and glucose molecules. Considering the sequence similarity with glycogenin, the glycosyltransferase domain of PDR1 may biosynthesize the polyglucan nanofilaments from UDP-glucose residues to form the PD ring filaments. OEM, outer envelope membrane; IMS, intermembrane space. (**C**) Schematic of *C. merolae* PDR1 and MDR1 domain structures. The glycosyltransferase domains of PDR1 and MDR1 identified them as type-8 subgroup members of the glycosyltransferase family. (**D**) Protein sequence similarities between PDR1, MDR1 and glycogenin-1. (**E**) Comparisons of the protein structure of glycogenin-1 (PDB: 1LL2) and the putative structures of the glycosyltransferase domains of PDR1 and MDR1. Orange arrows indicate specific insertion regions in the glycosyltransferase domains of PDR1 and MDR1. The protein structures of the PDR1 and MDR1 glycosyltransferase domains were modeled as described in Figure 2.

Although the molecular components of the outer PD ring have not yet been elucidated, despite more than 25 years passing since its discovery, a chemical-staining screen shed light on this issue [12]. Periodic acid-horseradish peroxidase staining indicated that the outer PD ring is likely to contain saccharic components, which led us to perform a proteomic analysis of the isolated plastid-division machinery fraction and identify a novel glycosyltransferase protein, PDR1 [12]. Interestingly, the expression of *PDR1* was specifically detected in the plastid-division phase, and PDR1 proteins assembled a single-ring structure at the plastid-division site. Ultrastructural studies clearly showed that PDR1 proteins localized on the outer PD ring filaments. Furthermore, analyses of the components of the purified PD ring filaments revealed that glucan molecules were components of the outer PD ring. PDR1 has sequence similarity to glycogenin, which acts as a priming protein for glycogen biosynthesis (Figure 4A) [53]; therefore, it is now hypothesized that PDR1 can elongate the glucan chain to biosynthesize PD ring filaments from UDP-glucose molecules, analogous to the biosynthesis of glycogen (Figure 4B). Taking these findings together, although the biosynthesis mechanism is still unclear, PDR1 is probably involved in the biosynthesis of the PD ring polyglucan filaments. Potential orthologs of *PDR1* have also been identified in land plant genomes. 

## 6. The Homology between Plastid- and Mitochondrial-Division Machinery

**Figure 5:**
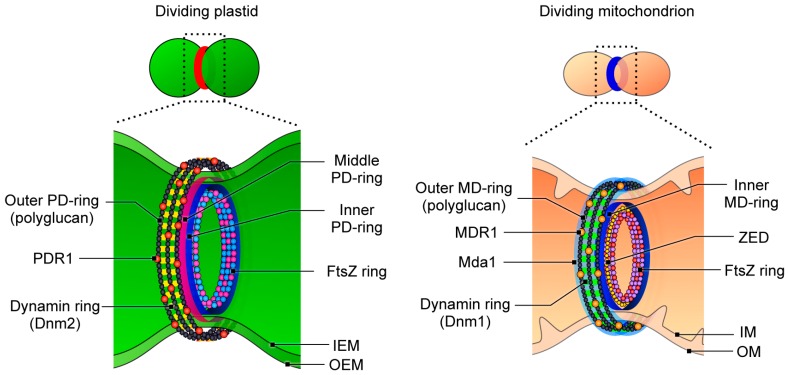
Schematic representations of the division machinery in plastids and mitochondria. IEM, inner envelope membrane; OEM, outer envelope membrane; IM, inner membrane; OM, outer membrane. For details on the mitochondrial-division machinery, see Refs. [1,54,55,56,57,58]. Modified from Yoshida et al. (2016) [21] and Yoshida et al. (2017) [54].

Over the past 25 years, the mode of plastid division has been unveiled and several key components of the division machinery have been identified. Furthermore, studies have revealed that the other endosymbiotic organelles, mitochondria, also proliferate via the activity of a supramolecular complex, the mitochondrial-division machinery, which, like the plastid-division machinery, comprises an electron-dense specialized ring called the mitochondrion-dividing (MD) ring which is the counterpart of the PD ring in mitochondrial division, the FtsZ ring, and a dynamin ring (Figure 5) [1,54,55]. Interestingly, some of the mechanisms of mitochondrial division are also very similar to those of the plastid-division machinery. In addition, a recent multi-omics analysis of isolated plastid- and mitochondrial-division machineries showed that 185 proteins, including 54 uncharacterized proteins, were present in the fraction, indicating that many unknown components may be involved in these machineries [54]. Indeed, a glycosyltransferase homologous to PDR1, MITOCHONDRION-DIVIDING RING1 (MDR1) (Figure 4C), was identified from these candidates, and a series of analyses showed that MDR1 is required for the assembly of the MD ring, which also consists of polyglucan filaments and is required for mitochondrial division [54]. Despite the low sequence similarity between PDR1 and MDR1 (Figure 4D,E), these proteins both have a glycosyltransferase domain belongs to the type-8 subgroup of the glycosyltransferase family and they have homologous functions in plastid and mitochondrial division [54]. Given that both plastids and mitochondria evolved from free-living bacteria, the compelling structural and mechanical similarity between the plastid- and mitochondrial-division machineries indicates that they were established in host cells to dominate and control the proliferation of these endosymbiotic organelles during their early evolution.

## 7. Conclusions and Perspectives

One of the current hot topics in this field is the elucidation of the system coordinating the cell-division cycle and the plastid/mitochondrial-division cycle [59,60,61]. Combinational analyses using genetic engineering and synchronized *C. merolae* cells has revealed the existence of a plastid-division checkpoint in the cell cycle, which is very likely to contribute to the permanent possession of plastids [19]; however, the fundamental mechanisms driving this process are still unclear. To identify the coordination system, the selection of suitable organisms for each analysis will be very important. The unicellular alga *C. merolae* and the land plant *A. thaliana* have enabled the molecular study of plastid division via their species-specific advantages. As a model organism, *A. thaliana* enabled many types of genetic studies to be conducted, leading to the identification of some of the genes responsible for plastid division (see for more details Chen et al. (2018) [6]). Studies in this area also benefit from using *C. merolae* cells, which can be synchronized to enable the isolation of intact plastid/mitochondrial-division machineries [13,54,62]. Furthermore, many genetic-engineering techniques have been recently established in *C. merolae* [18,63,64,65,66]. These innovations will enable dramatic advances in the investigation of the molecular mechanisms driving plastid division over the next decade; for example, an impressive recent study elucidated the crystal structure of the ARC6-PDV2 complex to reveal how protein-protein interactions translate information regarding plastid division across the double membrane, from the stromal region to the cytosol [17]. It is expected that the further identification of three-dimensional protein structures involved in the plastid-division machinery will open up a completely new avenue in the field.

## References

[1] Osteryoung K.W., Nunnari J. (2003). The division of endosymbiotic organelles. Science.

[2] Kuroiwa T., Misumi O., Nishida K., Yagisawa F., Yoshida Y., Fujiwara T., Kuroiwa H. (2008). Vesicle, mitochondrial, and plastid division machineries with emphasis on dynamin and electron-dense rings. Int. Rev. Cell Mol. Biol..

[3] Miyagishima S.Y., Nakanishi H., Kabeya Y. (2011). Structure, regulation, and evolution of the plastid division machinery. Int. Rev. Cell Mol. Biol..

[4] Yoshida Y., Miyagishima S.Y., Kuroiwa H., Kuroiwa T. (2012). The plastid-dividing machinery: Formation, constriction and fission. Curr. Opin. Plant Biol..

[5] Kuroiwa T., Kuroiwa H., Sakai A., Takahashi H., Toda K., Itoh R. (1998). The division apparatus of plastids and mitochondria. Int. Rev. Cytol..

[6] Chen C., MacCready J.S., Ducat D.C., Osteryoung K.W. (2018). The molecular machinery of chloroplast division. Plant Physiol..

[7] Miyagishima S.Y., Nishida K., Kuroiwa T. (2003). An evolutionary puzzle: Chloroplast and mitochondrial division rings. Trends Plant Sci..

[8] TerBush A.D., Yoshida Y., Osteryoung K.W. (2013). FtsZ in chloroplast division: Structure, function and evolution. Curr. Opin. Cell Biol..

[9] Miyagishima S.Y., Nishida K., Mori T., Matsuzaki M., Higashiyama T., Kuroiwa H., Kuroiwa T. (2003). A plant-specific dynamin-related protein forms a ring at the chloroplast division site. Plant Cell Online.

[10] Gao H., Kadirjan-Kalbach D., Froehlich J.E., Osteryoung K.W. (2003). ARC5, a cytosolic dynamin-like protein from plants, is part of the chloroplast division machinery. Proc. Natl. Acad. Sci. USA.

[11] Osteryoung K.W., Pyke K.A. (2014). Division and dynamic morphology of plastids. Annu. Rev. Plant Biol..

[12] Yoshida Y., Kuroiwa H., Misumi O., Yoshida M., Ohnuma M., Fujiwara T., Yagisawa F., Hirooka S., Imoto Y., Matsushita K. (2010). Chloroplasts divide by contraction of a bundle of nanofilaments consisting of polyglucan. Science.

[13] Yoshida Y., Kuroiwa H., Misumi O., Nishida K., Yagisawa F., Fujiwara T., Nanamiya H., Kawamura F., Kuroiwa T. (2006). Isolated chloroplast division machinery can actively constrict after stretching. Science.

[14] Glynn J.M., Yang Y., Vitha S., Schmitz A.J., Hemmes M., Miyagishima S.-Y., Osteryoung K.W. (2009). PARC6, a novel chloroplast division factor, influences FtsZ assembly and is required for recruitment of PDV1 during chloroplast division in Arabidopsis. Plant J..

[15] Zhang M., Chen C., Froehlich J.E., Terbush A.D., Osteryoung K.W. (2016). Roles of Arabidopsis PARC6 in coordination of the chloroplast division complex and negative regulation of FtsZ assembly. Plant Physiol..

[16] Glynn J.M., Froehlich J.E., Osteryoung K.W. (2008). Arabidopsis ARC6 coordinates the division machineries of the inner and outer chloroplast membranes through interaction with PDV2 in the intermembrane space. Plant Cell.

[17] Wang W., Li J., Sun Q., Yu X., Zhang W., Jia N., An C., Li Y., Dong Y., Han F. (2017). Structural insights into the coordination of plastid division by the ARC6-PDV2 complex. Nat. Plants.

[18] Sumiya N., Fujiwara T., Kobayashi Y., Misumi O., Miyagishima S.Y. (2014). Development of a heat-shock inducible gene expression system in the red alga *Cyanidioschyzon merolae*. PLoS ONE.

[19] Sumiya N., Fujiwara T., Era A., Miyagishima S.Y. (2016). Chloroplast division checkpoint in eukaryotic algae. Proc. Natl. Acad. Sci. USA.

[20] Sumiya N., Miyagishim S.Y. (2017). Hierarchal order in the formation of chloroplast division machinery in the red alga *Cyanidioschyzon merolae*. Commun. Integr. Biol..

[21] Yoshida Y., Mogi Y., TerBush A.D., Osteryoung K.W. (2016). Chloroplast FtsZ assembles into a contractible ring via tubulin-like heteropolymerization. Nat. Plants.

[22] Miyagishima S.Y., Takahara M., Mori T., Kuroiwa H., Higashiyama T., Kuroiwa T. (2001). Plastid division is driven by a complex mechanism that involves differential transition of the bacterial and eukaryotic division rings. Plant Cell.

[23] Miyagishima S.Y., Takahara M., Kuroiwa T. (2001). Novel filaments 5 nm in diameter constitute the cytosolic ring of the plastid division apparatus. Plant Cell.

[24] Sumiya N., Hirata A., Kawano S. (2008). Multiple FtsZ ring formation and reduplicated chloroplast DNA in *Nannochloris bacillaris* (Chlorophyta, Trebouxiophyceae) under phosphate-enriched culture. J. Phycol..

[25] Kuroiwa H., Mori T., Takahara M., Miyagishima S.Y., Kuroiwa T. (2002). Chloroplast division machinery as revealed by immunofluorescence and electron microscopy. Planta.

[26] Osteryoung K.W., Vierling E. (1995). Conserved cell and organelle division. Nature.

[27] Takahara M., Takahashi H., Matsunaga S.Y., Miyagishima S., Takano H., Sakai A., Kawano S., Kuroiwa T. (2000). A putative mitochondrial FtsZ gene is present in the unicellular primitive red alga *Cyanidioschyzon merolae*. Mol. Gen. Genet..

[28] Mori T., Kuroiwa H., Takahara M., Miyagishima S.Y., Kuroiwa T. (2001). Visualization of an FtsZ ring in chloroplasts of *Lilium longiflorum* leaves. Plant Cell Physiol..

[29] Vitha S., McAndrew R.S., Osteryoung K.W. (2001). FtsZ ring formation at the chloroplast division site in plants. J. Cell Biol..

[30] Löwe J., Amos L.A. (1998). Crystal structure of the bacterial cell-division protein FtsZ. Nature.

[31] Oliva M.A., Cordell S.C., Löwe J. (2004). Structural insights into FtsZ protofilament formation. Nat. Struct. Mol. Biol..

[32] Miyagishima S.Y., Nozaki H., Nishida K., Nishida K., Matsuzaki M., Kuroiwa T. (2004). Two types of FtsZ proteins in mitochondria and red-lineage chloroplasts: The duplication of FtsZ is implicated in endosymbiosis. J. Mol. Evol..

[33] Schmitz A.J., Glynn J.M., Olson B.J.S.C., Stokes K.D., Osteryoung K.W. (2009). Arabidopsis FtsZ2-1 and FtsZ2-2 are functionally redundant, but FtsZ-based plastid division is not essential for chloroplast partitioning or plant growth and development. Mol. Plant.

[34] Matsuzaki M., Misumi O., Shin-I T., Maruyama S., Takahara M., Miyagishima S.Y., Mori T., Nishida K., Yagisawa F., Nishida K. (2004). Genome sequence of the ultrasmall unicellular red alga *Cyanidioschyzon merolae* 10D. Nature.

[35] Eswar N., John B., Mirkovic N., Fiser A., Ilyin V.A., Pieper U., Stuart A.C., Marti-Renom M.A., Madhusudhan M.S., Yerkovich B. (2003). Tools for comparative protein structure modeling and analysis. Nucleic Acids Res..

[36] TerBush A.D., Osteryoung K.W. (2012). Distinct functions of chloroplast FtsZ1 and FtsZ2 in Z-ring structure and remodeling. J. Cell Biol..

[37] Shimada H., Koizumi M., Kuroki K., Mochizuki M., Fujimoto H., Ohta H., Masuda T., Takamiya K. (2004). ARC3, a chloroplast division factor, is a chimera of prokaryotic FtsZ and part of eukaryotic phosphatidylinositol-4-phosphate 5-kinase. Plant Cell Physiol..

[38] Maple J., Fujiwara M.T., Kitahata N., Lawson T., Baker N.R., Yoshida S., Møller S.G. (2004). GIANT CHLOROPLAST 1 is essential for correct plastid division in *Arabidopsis*. Curr. Biol..

[39] Shpetner H.S., Vallee R.B. (1989). Identification of dynamin, a novel mechanochemical enzyme that mediates interactions between microtubules. Cell.

[40] Praefcke G.J.K., McMahon H.T. (2004). The dynamin superfamily: Universal membrane tubulation and fission molecules?. Nat. Rev. Mol. Cell Biol..

[41] Bleazard W., McCaffery J.M., King E.J., Bale S., Mozdy A., Tieu Q., Nunnari J., Shaw J.M. (1999). The dynamin-related GTPase Dnm1 regulates mitochondrial fission in yeast. Nat. Cell Biol..

[42] Faelber K., Posor Y., Gao S., Held M., Roske Y., Schulze D., Haucke V., Noé F., Daumke O. (2011). Crystal structure of nucleotide-free dynamin. Nature.

[43] Ford M.G.J., Jenni S., Nunnari J. (2011). The crystal structure of dynamin. Nature.

[44] Sakaguchi E., Takechi K., Sato H., Yamada T., Takio S., Takano H. (2011). Three dynamin-related protein 5B genes are related to plastid division in *Physcomitrella patens*. Plant Sci..

[45] Miyagishima S.Y., Kuwayama H., Urushihara H., Nakanishi H. (2008). Evolutionary linkage between eukaryotic cytokinesis and chloroplast division by dynamin proteins. Proc. Natl. Acad. Sci. USA.

[46] Nishida K., Takahara M., Miyagishima S.Y., Kuroiwa H., Matsuzaki M., Kuroiwa T. (2003). Dynamic recruitment of dynamin for final mitochondrial severance in a primitive red alga. Proc. Natl. Acad. Sci. USA.

[47] Imoto Y., Kuroiwa H., Yoshida Y., Ohnuma M., Fujiwara T., Yoshida M., Nishida K., Yagisawa F., Hirooka S., Miyagishima S., Misumi O., Kawano S., Kuroiwa T. (2013). Single-membrane-bounded peroxisome division revealed by isolation of dynamin-based machinery. Proc. Natl. Acad. Sci. USA.

[48] Marchler-Bauer A., Derbyshire M.K., Gonzales N.R., Lu S., Chitsaz F., Geer L.Y., Geer R.C., He J., Gwadz M., Hurwitz D.I. (2015). CDD: NCBI’s conserved domain database. Nucleic Acids Res..

[49] Kuroiwa T. (1998). The primitive red algae *Cyanidium caldarium* and *Cyanidioschyzon merolae* as model system for investigating the dividing apparatus of mitochondria and plastids. BioEssays.

[50] Hashimoto H. (1986). Double ring structure around the constricting neck of dividing plastids of *Avena sativa*. Protoplasma.

[51] Miyagishima S.Y., Itoh R., Toda K., Takahashi H., Kuroiwa H., Kuroiwa T. (1998). Identification of a triple ring structure involved in plastid division in the primitive red alga *Cyanidioschyzon merolae*. J. Electron Microsc..

[52] Kuroiwa H., Mori T., Takahara M., Miyagishima S.Y., Kuroiwa T. (2001). Multiple FtsZ rings in a pleomorphic chloroplast in embryonic cap cells of *Pelargonium zonale*. Cytologia.

[53] Lomako J., Lomako W.M., Whelan W.J. (2004). Glycogenin: The primer for mammalian and yeast glycogen synthesis. Biochim. Biophys. Acta.

[54] Yoshida Y., Kuroiwa H., Shimada T., Yoshida M., Ohnuma M., Fujiwara T., Imoto Y., Yagisawa F., Nishida K., Hirooka S. (2017). Glycosyltransferase MDR1 assembles a dividing ring for mitochondrial proliferation comprising polyglucan nanofilaments. Proc. Natl. Acad. Sci. USA.

[55] Kuroiwa T., Nishida K., Yoshida Y., Fujiwara T., Mori T., Kuroiwa H., Misumi O. (2006). Structure, function and evolution of the mitochondrial division apparatus. Biochim. Biophys. Acta.

[56] Van der Bliek A.M., Shen Q., Kawajiri S. (2013). Mechanisms of mitochondrial fission and fusion. Cold Spring Harb. Perspect. Biol..

[57] Friedman J.R., Nunnari J. (2014). Mitochondrial form and function. Nature.

[58] Roy M., Reddy P.H., Iijima M., Sesaki H. (2015). Mitochondrial division and fusion in metabolism. Curr. Opin. Cell Biol..

[59] Kobayashi Y., Kanesaki Y., Tanaka A., Kuroiwa H., Kuroiwa T., Tanaka K. (2009). Tetrapyrrole signal as a cell-cycle coordinator from organelle to nuclear DNA replication in plant cells. Proc. Natl. Acad. Sci. USA.

[60] Kobayashi Y., Imamura S., Hanaoka M., Tanaka K. (2011). A tetrapyrrole-regulated ubiquitin ligase controls algal nuclear DNA replication. Nat. Cell Biol..

[61] Miyagishima S.Y., Fujiwara T., Sumiya N., Hirooka S., Nakano A., Kabeya Y., Nakamura M. (2014). Translation-independent circadian control of the cell cycle in a unicellular photosynthetic eukaryote. Nat. Commun..

[62] Suzuki K., Ehara T., Osafune T., Kuroiwa H., Kawano S., Kuroiwa T. (1994). Behavior of mitochondria, chloroplasts and their nuclei during the mitotic cycle in the ultramicroalga *Cyanidioschyzon merolae*. Eur. J. Cell Biol..

[63] Ohnuma M., Yokoyama T., Inouye T., Sekine Y., Tanaka K. (2008). Polyethylene glycol (PEG)-mediated transient gene expression in a red alga, *Cyanidioschyzon merolae* 10D. Plant Cell Physiol..

[64] Ohnuma M., Misumi O., Fujiwara T., Watanabe S., Tanaka K., Kuroiwa T. (2009). Transient gene suppression in a red alga, *Cyanidioschyzon merolae* 10D. Protoplasma.

[65] Fujiwara T., Kanesaki Y., Hirooka S., Era A., Sumiya N., Yoshikawa H., Tanaka K., Miyagishima S.Y. (2015). A nitrogen source-dependent inducible and repressible gene expression system in the red alga *Cyanidioschyzon merolae*. Front. Plant Sci..

[66] Fujiwara T., Ohnuma M., Kuroiwa T., Ohbayashi R., Hirooka S., Miyagishima S.Y. (2017). Development of a double nuclear gene-targeting method by two-step transformation based on a newly established chloramphenicol-selection system in the red alga *Cyanidioschyzon merolae*. Front. Plant Sci..

